# Knowledge and attitude of secondary school students in Nakaseke, Uganda towards HIV transmission and treatment

**DOI:** 10.12688/aasopenres.13210.1

**Published:** 2021-05-05

**Authors:** Patricia Nabisubi, Stephen Kanyerezi, Grace Kebirungi, Gerald Mboowa

**Affiliations:** 1 Department of Bioinformatics, The African Center of Excellence in Bioinformatics and Data-Intensive Sciences, the Infectious Disease Institute, Makerere University, Kampala, Central, 256, Uganda; 2 Department of Immunology and Molecular Biology, College of Health Sciences, Makerere University, Kampala, Central, 256, Uganda

**Keywords:** Attitude, Knowledge, HIV/AIDS, School, Transmission, Treatment, Uganda

## Abstract

**Background:** One of the major health concerns in Nakaseke district, Uganda is the high prevalence of HIV/AIDS. According to the Joint United Nations Programme on HIV/AIDS (UNAIDS), as of March 2014, the prevalence rate of the disease in the district was estimated at about 8%, compared to the national average of 6.5%, making Nakaseke district have the sixth-highest prevalence rate of HIV/AIDS in the entire country. We set out to explore the knowledge and attitude of secondary school students in Nakaseke, Uganda on HIV transmission and treatment.

**Methods: **This was a cross sectional survey-based study with data collected during the month of February 2020. Data were analyzed using R programming language version 3.6.2.

**Results: **A total of 163 participants volunteered for the study, 53.37% males and 46.63% females with ages ranging from 12 – 20 years. Participants came from 5 senior classes (S1, S2, S3, S4 and S6). In total, 87.73% participants were aware of HIV/AIDS while 12.27% were not. The major source of information was through teachers/schools. 96.50% knew the mode of transmission of HIV/AIDS and 95.11% were conversant with HIV/AIDS prevention. 63.6% were aware of the terms DNA and genes whereas 36.36% were not.

**Discussion: **Generally, the students in Nakaseke district, Uganda had a high level of awareness of HIV/AIDS. However, with regards to aspects such as the cause and modern prevention methods like taking prep and prevention of mother to child transmission were less known to them. Efforts to find a cure for HIV/AIDS are still in vain. Therefore, strong emphasis on up to date control and prevention methods should be implemented to fight the HIV/AIDS scourge **.**

## Introduction

The vast majority of people living with human immunodeficiency virus (HIV) are located in low- and middle- income countries, with an estimated 68% living in sub-Saharan Africa ^1^. Among this group, 20.6 million are living in East and Southern Africa which saw 800,000 new HIV infections in 2018 ^1^. HIV and acquired immunodeficiency syndrome (HIV/AIDS) has remained a challenge in Uganda among adolescents despite the ABC (Abstinence, Be faithful, use a Condom) strategy ^2^. Globally, the most vulnerable group of individuals to HIV infection are reported to be the youth in the reproductive age group of 15 – 24 years with adolescents contributing to a large percentage ^2– 4^. Previous research found that only 45.5% of women and men between 15–24 years old correctly identified ways of preventing the transmission of HIV through sex ^5^. In 2018, Uganda had 53,000 people newly infected with HIV ^5^. There are many political and cultural barriers which have hindered effective HIV prevention programming in Uganda. Consequently, new HIV infections are expected to rise in coming years ^6^.

Community-based interventions (CBIs) for the prevention and control of HIV allow increased access and ease availability of medical care to populations at risk, or already infected with HIV by reaching individuals in schools, homes or community centers. School-based delivery of HIV prevention education have also been advocated as potential strategies to target high-risk youth groups ^7^. Creating awareness among the youth on HIV/AIDS is the key to reducing its spread.

The aim of this study was to evaluate the knowledge and attitude of secondary school students towards HIV transmission and treatment in a rural secondary school in Nakaseke, Uganda.

## Methods

### Study design and setting

**Figure 1.**
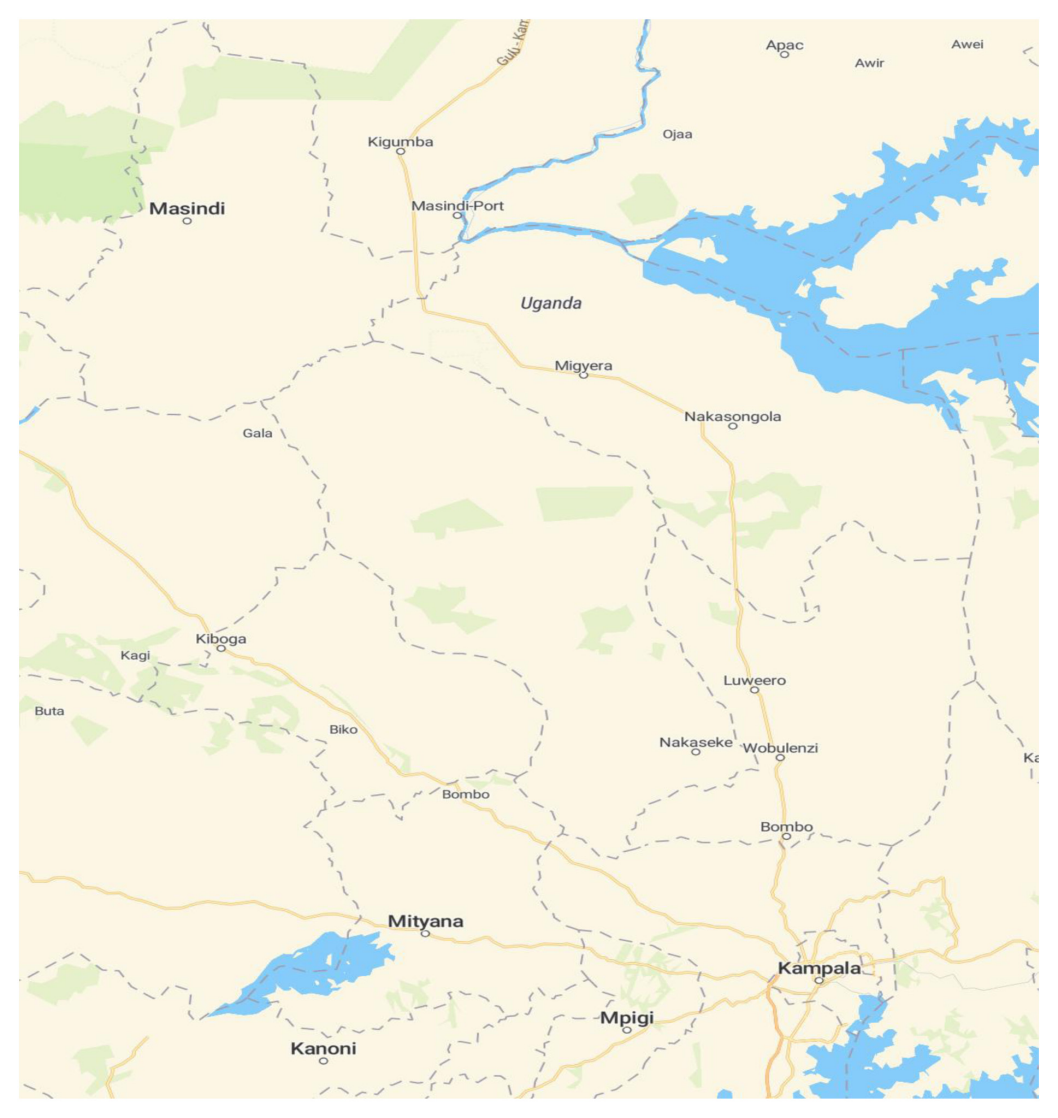
Map of Nakaseke district and its borders (Source: https://www.maptiler.com/maps).

This was a cross-sectional study conducted in a secondary school in Nakaseke district in Uganda. Nakaseke district was conveniently chosen to represent a rural setting. It was purposely selected because it had the sixth-highest prevalence rate of HIV/AIDS in 2014 in Uganda. Nakaseke district is bordered by Nakasongola district to the north ( Figure 1). The location of the district headquarters lies approximately 66 kilometers (41 mi) by road, north of Kampala, the capital of Uganda and the largest city in the country. It is estimated that 59.2% of the Nakaseke district community is literate, which is largely limited to the local Luganda language. This district has seven health units including a 100-bed public hospital, Nakaseke Hospital, administered by the Uganda Ministry of Health. Nakaseke Hospital is connected to other health units by a radio ^8^. One of the major health concerns is the high prevalence of HIV/AIDS. The prevalence rate of the disease in the district was estimated at about 8%, compared to the national average of 6.5%. Nakaseke district has the sixth-highest prevalence rate of HIV/AIDS in Uganda ^9^.

### Study population and sampling procedures

The sampling procedure was voluntary. Students from classes S1, S2, S3, S4, and S6 were interviewed. S5 students were excluded because they had not yet reported to school. The study was expected to interview a total of 200 students but only 163 of them turned up.

### Data collection

The students were interviewed using structured questionnaires ^22^ adopted from 10, with the help of trained research assistants in the students’ language of preference (English or Luganda). The questionnaire had closed ended questions, presented in two formats: yes or no, and multiple choice or objective responses with an aim of accessing the knowledge of students about HIV/AIDS and their attitudes towards HIV transmission, prevention and stigma.

### Study variables

Treatment, transmission, prevention and stigma were the variables used to evaluate the degree of knowledge about HIV. This was done through computing percentages for every correct response and percentages greater than 50 qualified as good knowledge about HIV. The demographic characteristics: age, gender and class were also considered.

### Data quality control

Trained research assistants interviewed the participants in their preferred language of communication (English or Luganda) to ensure comprehension and better expression while responding to the questions. Questions were simple and easy to understand. The data was entered using an excel spreadsheet, triple checked for errors, saved as a comma-separated values file (CSV) and later imported into R.

### Data analysis plan

All questions in the questionnaires were entered into the excel spreadsheet and the data was imported and analyzed using R version 3.6.2. All questionnaires with missing data were regarded as invalid and excluded from the analysis. The frequencies and percentage responses for each question was computed and the data was transformed into tables.

### Ethical considerations

Ethical clearance and approval for project was obtained from the School of Medicine Research and Ethics Committee (SOMREC) at Makerere University College of Health Sciences with approval number, #REC REF 2019–028. Administrative permission was obtained from both the Ugandan Ministry of Education and Sports (assigned reference number, ADM. 217/323/01) as well as the school. For participants in the age group 12 to 17 years, consent was sought from the parents/guardians and then assent was obtained from the student. For mature and emancipated minors, written informed consent was independently sought from them. For the age group of 18 years and above, consent was sought from the students themselves. Confidentiality of information was ensured by the principal investigator during and after the study by blinding the participants’ names and replacing them with arbitrarily chosen IDs.

## Results

163 participants volunteered for the study with more males (87, 53.37%) than females (76, 46.63%) ^21^. The participants were aged between 12 – 20 years, with a modal age group of 15 – 17 years and an average age of 15.45 ± 1.82.

In total, 143 (87.73%) participants were aware of HIV/AIDS while 20 (12.27%) were not. The mean age of those who were aware of the disease was 15.37 ± 1.77 years whereas those who were not had a mean age of 16.05 ± 2.06 years. The downstream analysis after the question regarding awareness of HIV/AIDS was exclusive of participants that were not aware of the disease. Most of the participants (109, 76.22%) were aware of HIV treatment (antiretroviral drugs; ARVs) while 34 (23.78%) were not aware ( Table 2). In total, 65 (45.46%) knew that HIV can be transmitted through sexual intercourse with an HIV infected person without using a condom, 5 (3.50%) mentioned sharing injecting equipment, and 68 (47.56%) had multiple responses (contaminated blood transfusion or organs/tissues, sexual intercourse with an HIV infected person without using a condom, sharing injecting equipment and mother to child transmission during childbirth and breastfeeding). Only 5 (3.50%) participants didn’t know of any mode of transmission ( Table 4). Blood (65, 45.64%) was the most known bodily fluid in which HIV is transmitted. Others mentioned breastmilk (1, 0.70%), vaginal fluids (4, 2.80%) and 33 (23.08%) participants did not know ( Table 5).

**Table 1.** Demographic characteristics of participants.

Demographic characteristics	Response item	Frequency	Percentage %
	Male	87	53.37
**Gender**			
	Female	76	46.63
**Total**		**163**	**100.00**
	12	4	2.45
	13	16	9.82
	14	40	24.54
	15	28	17.18
**Age Distribution**			
	16	31	19.02
	17	16	9.82
	18	19	11.66
	19	7	4.29
	20	2	1.23
**Total**		**143**	**100.00**

**Table 2.** Response on knowledge about HIV/AIDS. ARVs=antiretroviral drugs.

Response item	Response	Frequency	Percentage
	Yes	143	87.73
**Are you aware of HIV/AIDS**			
	No	20	12.27
**Total**		**163**	**100.00**
	Yes	109	76.22
**Are you aware of ARVs**			
	No	34	23.78
**Total**		**143**	**100.0**

**Table 3.** Sources of information (multiple responses).

Response item	Response	Frequency	Percentage
	1) Church or Mosque	2	1.40
	2) Family or Friends	20	13.99
	3) Health professionals	29	20.28
	4) TV or Radio	22	15.39
	5) Teachers or schools	31	21.68
	2 and 4	7	4.90
**From which sources** **have you heard** **about HIV/AIDS**			
	2 and 5	5	3.50
	2 and 3	4	2.80
	2, 3 and 5	5	3.50
	3 and 4	3	2.10
	3 and 5	15	10.50
**Total**		**143**	**100.00**

**Table 4.** Response on modes of HIV/AIDS transmission.

Response item	Response	Frequency	Percentage
	1) Sexual intercourse without a condom	65	45.46
	2) Share injecting equipment	5	3.50
	3) Do not know	5	3.50
**How is HIV** **transmitted**	4) 2 and Contaminated blood transfusion	1	0.70
	5) 1 and Contaminated blood transfusion	4	2.80
	2 and 5	2	1.40
	2, 5 and mother to child transmission	27	18.88
	2 and 5	34	23.78
**Total**		**143**	**100.00**

**Table 5.** Response on bodily fluids that contain HIV virus.

Response item	Response	Frequency	Percentage
	1) Blood	65	45.46
	2) Breast milk	1	0.70
	3) Vaginal Fluids	4	2.80
	4) Do not know	33	23.08
	1 and 2	8	5.59
	1 and semen	3	2.10
**Which bodily** **fluids contain HIV**	1, 2 and semen	2	1.40
	1, semen and rectal fluids or anal mucous	3	2.10
	1, 3 and semen	2	1.40
	1, 2, 3 and semen	8	5.59
	1, 2 and 3	5	3.50
	1 and 3	9	6.29
**Total**		**143**	**100.00**

In regards to the cause of HIV/AIDS, most of the participants (88, 61.54%) knew that it was caused by a virus, 9 (6.29%) said that it was caused by bacteria and the rest 46 (32.17%) didn’t know the cause ( Table 6). For the signs and symptoms of HIV/AIDS, 105 (73.43%) individuals pointed out frequent illnesses and 38 (26.57%) did not know the signs and symptoms one could identify a person infected with HIV/AIDS. 85 (59.44%) participants had ever seen someone with HIV/AIDS whereas the rest 58 (40.56%) had never. Among those that had ever seen someone infected with the disease, 52 (61.18%) had guessed, 26 (30.59%) were told by the patient, 3 (3.53%) got to know through their families, 3 (3.53%) knew through the hospitals and 1 (1.18%) got to know through their teachers/schools ( Table 7).

**Table 6.** Response to the cause of HIV/AIDS.

Response item	Response	Frequency	Percentage
	Virus	88	61.54
**What is the cause** **of HIV/AIDS**	Bacteria	9	6.29
	Do not Know	46	32.17
**Total**		**143**	**100.0**

**Table 7.** Distribution of knowledge about HIV/AIDS patient.

Response item	Response	Frequency	Percentage
	Frequent illness	105	73.43
**Signs and symptoms**			
	Do not Know	38	26.57
**Total**		**143**	**100.0**
	Yes	85	59.44
**Have you ever seen HIV** **patient**			
	No	58	40.56
**Total**		**143**	**100.0**
	Guessed	52	61.18
	Hospital	3	3.53
**If Yes, how did you Know**	Teachers/Schools	1	1.18
	Patient told you	26	30.59
	Family	3	3.53
**Total**		**85**	**100.01**

When participants were asked whether there was cure for HIV, 116 (81.12%) knew that there was no cure for HIV whereas 27 (18.88%) thought that there was cure for HIV. Those that said there is a cure for HIV claimed to have heard from different sources of information. 5 (18.52%) from health professionals, 5 (18.52%) from friends/family, 8 (29.63%) from TV/Radio, 6 (22.22%) from Teachers/school, 2 (7.41%) from church/schools and 1 (3.71%) from both health professionals and TV/ Radio. None got to know from the internet and newspapers ( Table 8). HIV prevention methods were generally known. Most responses (52, 36.36%) were for appropriate and consistent condom use, 48 (33.57%) were for abstinence, 2 (1.40%) for avoiding sharing sharp objects and the rest (1, 0.70%) were for taking prep consistently, checkups and avoiding blood contact with an infected person ( Table 9). Most of the participants (126, 88.11%) knew that HIV/AIDS was diagnosed using a blood test, 1 (0.70%) said that it was diagnosed through the urine test and 16 (11.19%) did not know how the disease is diagnosed ( Table 10).

**Table 8.** Response to the HIV cure presence.

Response item	Response	Frequency	Percentage
	Yes	27	18.88
**Is there cure for HIV**			
	No	116	81.12
**Total**		**143**	**100.00**
	Church or Mosque	2	7.41
	Family or Friends	5	18.52
	Health Professionals	5	18.52
**If yes, how did you** **know about it**			
	Radio or TV	8	29.63
	Teachers or schools	6	22.22
	Health professionals and TV	1	3.71
**Total**		**27**	**100.00**

**Table 9.** Response to knowledge about HIV/AIDS prevention strategy.

Response item	Response	Frequency	Percentage
	1) Abstain	48	33.57
	2) Avoid sharing sharp objects	2	1.40
	3) Taking Prep or treatment consistently	1	0.70
	4) Checkups	1	0.70
	5) Do not know	7	4.90
**How do you prevent** **getting or transmitting** **HIV/AIDS**			
	6) Appropriate condom use	52	36.36
	7) Avoid blood contact with infected person	1	0.70
	1 and 2	12	8.39
	1, 2 and 6	1	0.70
	1 and Testing for HIV/AIDS	1	0.70
	1 and 6	11	7.69
	2 and 6	6	4.20
**Total**		**143**	**100.00**

**Table 10.** Response on knowledge about HIV diagnosis.

Response item	Response	Frequency	Percentage
	Blood test	126	88.11
**How is HIV diagnosed**	Urine test	1	0.70
	Don’t know	16	11.19
**Total**		**143**	**100.00**

With regards to knowledge on HIV transmission from parent to child, the students’ responses were generally not in line with the scientific facts ( Table 11). Over two thirds (99, 69.23%) of students said they would not marry a person infected with HIV and 92 (64.34%) would discontinue the relationship if they discovered that the person they were dating was infected with HIV. Though, 44 (30.77%) respondents were willing to marry a person infected with HIV, 9 (6.29%) would continue the relationship and 28 (19.58%) would seek counselling and treatment ( Table 12).

**Table 11.** Response on knowledge of HIV infected parent to child transmission.

Response item	Response	Frequency	Percentage
	All	25	17.48
	Half	20	13.99
**One parent infected**	Quarter	5	3.50
	None	79	55.24
	Do not know	14	9.79
**Total**		**143**	**100.00**
	All	53	37.06
**All parents infected**	Half	11	7.69
	None	60	41.96
	Do not know	19	13.29
**Total**		**143**	**100.00**

**Table 12.** Response on knowledge about dealing with HIV partners.

Response item	Response	Frequency	Percentage (%)
	Yes	44	30.77
**Can you marry an** **HIV patient**			
	No	99	69.23
**Total**		**143**	**100.00**
	1) Continue with the relationship	9	6.29
**What happens when** **you discover you are** **dating an HIV patient**	2) Discontinue the relationship	92	64.34
	3) Seek counselling and treatment	28	19.58
	4) Do not know	3	2.10
	1 and 3	7	4.9
	2 and 3	4	2.80
**Total**		**143**	**100.00**

Concerning knowledge on Host Genetics in HIV infection, 91 (63.64%) students had heard about the terms “DNA” or “Genes” and 52 (36.36%) hadn’t. For those who had heard about the terms, their major sources of information included; TV or radio (33, 36.26%), health professionals (29, 31.87%), family/friends (11, 12.09%), teachers/schools (11, 12.09%), church/mosque (1, 1.10%) and movies (1, 1.10%). Those who knew about DNA or genes were further asked whether their DNA or genes would determine if they would get infected with HIV or not; 56 (61.54%) said no, 31 (34.07%) said yes and 4 (4.40%) did not know. In addition, they were also asked whether their DNA or Genes would affect the outcome of their HIV treatment; 60 (65.93%) responded no to the question, 26 (28.57%) knew that DNA would affect HIV treatment whereas 5 (5.50%) were undecided ( Table 13).

**Table 13.** Response on knowledge on host genetics in HIV infection.

Response item	Response	Frequency	Percentage (%)
	Yes	91	63.64
**Aware of the term DNA/genes**			
	No	52	36.36
**Total**		**143**	**100.00**
	1) Church or Mosque	1	1.10
	2) Friends or Family	11	12.09
	3) Health professionals	29	31.87
**If yes, how did you hear them**	4) Teachers or schools	11	12.09
	5) TV or Radio	33	36.26
	6) Movies	1	1.10
	2 and 5	2	2.20
	2 and 3	1	1.10
	3 and 5	1	1.10
	4 and 5	1	1.10
**Total**		**91**	**100.00**
	Yes	31	34.07
**Is HIV infection DNA determined**			
	No	56	61.54
	Do not know	4	4.40
**Total**		**91**	**100.00**
	Yes	26	28.57
**Does your DNA determine the** **outcome of your HIV treatment**			
	No	60	65.93
	Do not know	5	5.50
**Total**		**91**	**100.00**

## Discussion

### Demographic characteristics

The study assessed the knowledge, attitudes and perception of secondary school students in Nakaseke, Uganda. Of the 163 participants, 143 (87.73%) were aware of HIV/AIDS and 20 (12.27%) were not. The mean age of those who were aware of the disease was 15.37 ± 1.77 years while those who were not aware of the disease had a mean age of 16.05 ± 2.06. Similar results were seen in 11 and 12

### Knowledge and attitude about HIV/AIDS diagnosis, symptoms, transmission, prevention, treatment, and cure by participants in a rural school

Since awareness hinges on the knowledge about HIV/AIDS, what our participants knew about HIV was very key. The source of information about HIV was also important as it conveyed the basic information about HIV/AIDS and in this study. Teachers or schools (21.68%), similar to 13 and health professionals (20.28%) were the most used avenues to convey the message to the participants. The Presidential Initiative on AIDS Strategy to Youth (PIASCY) started in 2001, and was introduced in all primary and post primary schools’ education curriculum ^14^. This program was designed to prevent the spread of HIV/ AIDS and to mitigate its impact on primary and post-primary education institutions in Uganda. It is no surprise that the participants received most information about HIV/AIDS through their teachers ^20^. None of the participants got information through the internet and newspapers. This is typical of rural secondary schools as such services do not reach rural areas ^15^. A substantial number of students (87.73%) were aware of HIV/AIDS and its treatment (76.22%) except for a few. The bodily fluids through which HIV is spread were also known, with blood taking the biggest share (45.46%) hence being in concordance with 16 and 17. In spite of that, there were some misconceptions where some students thought HIV is spread through saliva, tears, vomitus and pus. These need to be addressed as they can cause stigma and discrimination towards patients with HIV.

A small number of students (18.88%) thought HIV/AIDS is curable. They might have mistaken the available drugs for having a curative effect. To remedy this, more emphasis should be put on the microorganism that causes HIV because then, they would know the gravity of the disease and that it is incurable. Quite a number of students (32.17%) didn’t know what causes HIV/AIDS and others (6.29%) thought HIV/AIDS was caused by bacteria. Concerning the signs and symptoms of HIV/AIDS, several students knew them, showing that HIV/AIDS is not a rare disease. Most (61.18%) have known that someone has HIV/AIDS through guessing, 30.59% told by a patient, 3.53% told by family, and 3.53% have seen them in hospitals. Although there are several means through which students have known peoples’ HIV status, it is unethical for health practitioners to disclose a patient’s status as his/her confidentiality is violated ^18^. In this modern era, ARVs have made it increasingly difficult to identify someone living with HIV/AIDS. It’s quite understandable if someone cannot tell whether a person is infected with HIV or not. Therefore, people should be encouraged to take an HIV/AIDS test despite the way someone looks.

Despite a vast knowledge on HIV/AIDS prevention like abstinence (33.57%), appropriate and consistent use of a condom (36.36%) to mention but a few, modern methods like taking prep (0.70%) in case of accidental infection or rape and mother to child transmission were less known to students. This was reflected by the few number of students that mentioned taking prep and their responses concerning a child’s chances of getting HIV/AIDS if either or both of the parents were infected. There was a difference in the responses to chances of a child getting HIV/AIDS if only one or both parents were infected showing that the students do not quite know that a child can be born without HIV/AIDS irrespective of the parents’ HIV status. It is important to sensitize the population on the recent advances in HIV/AIDS such as those mentioned above as they greatly help to control transmission of the disease and thus reduce the number of people infected with HIV/AIDS ^10^. Most participants were not willing to marry someone infected with HIV however there was a small percentage (4.90%) of people that were willing to continue the relationship and seek counseling and treatment. Nonetheless, even those (2.80%) that would discontinue the relationship were willing to encourage their partner to seek counseling and treatment. Despite that, there was still a huge number (64.34%) of people that didn’t want to have a relationship with someone with HIV. Therefore, there is still a lot of work needed to be done to change the attitude of these people towards HIV patients due to limited knowledge about HIV/AIDs in schools in remote areas.

### Knowledge and attitude about the role of an individual’s genetics on HIV/AIDS in acquisition and effect on treatment

More than half (63.64%) of the total number of participants were familiar with the terms DNA and genes. However, their knowledge of the terms was in respect to paternity tests rather than the role they play in acquisition and their effect on HIV/AIDS. This is not alarming because the basics of genomics are not taught till the advanced level in secondary school.

### Limitations of the study

Having carried out the study during the time senior five-students hadn’t resumed school, it reduced the classes interviewed thus affecting our study indirectly. Inaccurate responses from some participants could have limited the study as well. This would have been due to their fear to disclose what they knew

## Conclusion

This study provides preliminary data from a country and region where current information on the knowledge of young adults about HIV/AIDS and their attitude toward infected persons are sparse. This study highlights the basic knowledge of HIV/AIDS among young students, modes of transmission, treatment and management; it also indicates that stigma about the disease and discrimination of affected individuals in society is common among students. The basic approach for control and prevention of HIV/AIDS remains prevention through better knowledge and awareness since an effective cure or vaccine is not yet available.

Generally, the secondary school students had a high level of awareness of HIV/AIDS. However, with regards to aspects such as the cause and modern prevention methods like taking prep and prevention of mother to child transmission were less known to them. Efforts to find a cure for HIV/AIDS are still in vain. Therefore, strong emphasis on up to date control and prevention methods should be implemented to fight the HIV/AIDS scourge.

## Data availability

### Underlying data

Figshare: N_Raw_data.xlsx. https://doi.org/10.6084/m9.figshare.14399453 ^21^.

This project contains the following underlying data:

- N_Raw_data.xlsx (Raw survey data)

### Extended data

Figshare: Questionnaire.docx. https://doi.org/10.6084/m9.figshare.14399393 ^22^.

This project contains the following extended data:

- Questionnaire.docx

Data is available under terms of the Creative Commons Zero "No rights reserved" data waiver (CC0 1.0 Public domain dedication).
