# Knowledge and attitude of secondary school students in Nakaseke, Uganda towards HIV transmission and treatment

**DOI:** 10.12688/aasopenres.13210.2

**Published:** 2021-07-12

**Authors:** Patricia Nabisubi, Stephen Kanyerezi, Grace Kebirungi, Gerald Mboowa

**Affiliations:** 1 Department of Bioinformatics, The African Center of Excellence in Bioinformatics and Data-Intensive Sciences, the Infectious Diseases Institute, Makerere University, Kampala, Central, 256, Uganda; 2 Department of Immunology and Molecular Biology, College of Health Sciences, Makerere University, Kampala, Central, 256, Uganda

**Keywords:** Attitude, Knowledge, HIV/AIDS, School, Transmission, Treatment, Uganda

## Abstract

**Background:** One of the major health concerns in Nakaseke district, Uganda is the high prevalence of HIV/AIDS. According to the Joint United Nations Programme on HIV/AIDS (UNAIDS), as of March 2014, the prevalence rate of the disease in the district was estimated at about 8%, compared to the national average of 6.5%, making Nakaseke district have the sixth-highest prevalence rate of HIV/AIDS in the entire country. We set out to explore the knowledge and attitude of secondary school students in Nakaseke, Uganda on HIV transmission and treatment.

**Methods: **This was a cross sectional survey-based study with data collected during the month of February 2020. Data were analyzed using R programming language version 3.6.2.

**Results: **A total of 163 participants volunteered for the study, 53.37% males and 46.63% females with ages ranging from 12 – 20 years. Participants came from 5 senior classes (S1, S2, S3, S4 and S6). In total, 87.73% participants were aware of HIV/AIDS while 12.27% were not. The major source of information was through teachers/schools. 96.50% knew the mode of transmission of HIV/AIDS and 95.11% were conversant with HIV/AIDS prevention. 63.6% were aware of the terms DNA and genes whereas 36.36% were not.

**Discussion: **Generally, the students in Nakaseke district, Uganda had a high level of awareness of HIV/AIDS based on Bloom’s cut-off point. However, with regards to aspects such as the cause and modern prevention methods like taking prep and prevention of mother to child transmission were less known to them. Efforts to find a cure for HIV/AIDS are still in vain. Therefore, strong emphasis on up to date control and prevention methods should be implemented to fight the HIV/AIDS scourge **.**

## Introduction

The vast majority of people living with human immunodeficiency virus (HIV) are located in low- and middle- income countries, with an estimated 68% living in sub-Saharan Africa ^1^. Among this group, 20.6 million are living in East and Southern Africa which saw 800,000 new HIV infections in 2018 ^1^. HIV and acquired immunodeficiency syndrome (HIV/AIDS) has remained a challenge in Uganda among adolescents despite the ABC (Abstinence, Be faithful, use a Condom) strategy ^2^. Globally, the most vulnerable group of individuals to HIV infection are reported to be the youth in the reproductive age group of 15 – 24 years with adolescents contributing to a large percentage ^3– 5^. Previous research found that only 45.5% of women and men between 15–24 years old correctly identified ways of preventing the transmission of HIV through sex. In 2018, Uganda had 53,000 people newly infected with HIV ^6^. There are many political and cultural barriers which have hindered effective HIV prevention programming in Uganda. Consequently, new HIV infections are expected to rise in coming years ^7^.

Community-based interventions (CBIs) for the prevention and control of HIV allow increased access and ease availability of medical care to populations at risk, or already infected with HIV by reaching individuals in schools, homes or community centers. School-based delivery of HIV prevention education has also been advocated as potential strategies to target high-risk youth groups ^8, 9^. Community engagement activities with Persons Living with HIV in Nakaseke have been utilised to reduce the number of new infections. This is led by local leaders, the sub county chief, religious leaders, Village health team members, health workers, community decision makers and the community development officers ^10^. But this is largely targeting adults while students at schools may get HIV/AIDS prevention messages either through their parents, teachers, fellow students and the Presidential Initiative on AIDS Strategy to Youth (PIASCY). Creating awareness among the youth on HIV/AIDS is the key to reducing its spread.

The aim of this study was to evaluate the knowledge and attitude of secondary school students towards HIV transmission and treatment in a rural secondary school in Nakaseke, Uganda.

## Methods

### Study design and setting

**Figure 1.**
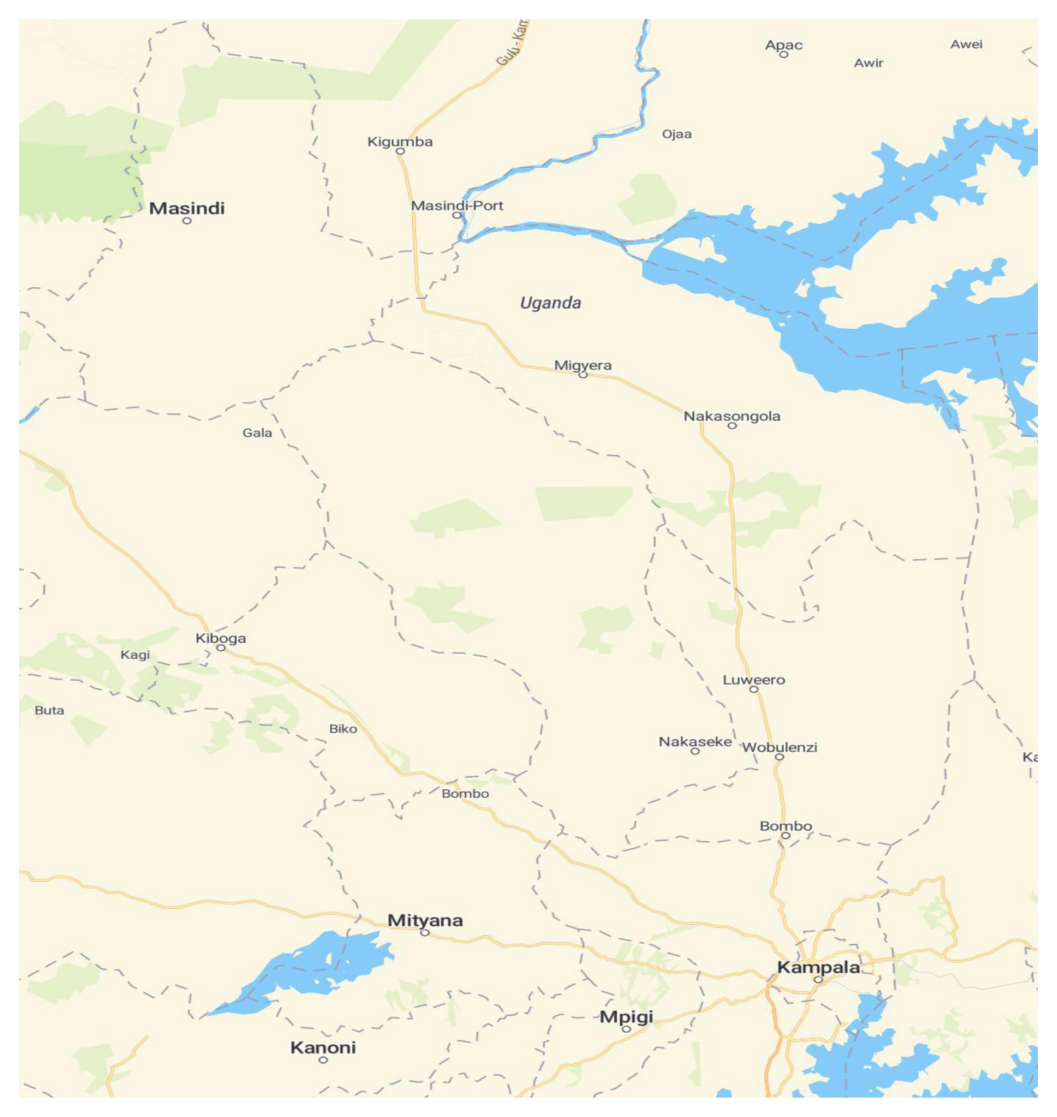
Map of Nakaseke district and its borders (Source: https://www.maptiler.com/maps).

This was a cross-sectional study conducted in a secondary school in a church of Uganda founded school in Nakaseke district of Uganda. This school is both day and boarding and students are supported through a number of mechanisms such as the government of Uganda’s universal secondary education quota system, while others are sponsored privately or by non-governmental organisations like World Vision Uganda. Nakaseke district was conveniently chosen to represent a rural setting. It was purposely selected because it had the sixth-highest prevalence rate of HIV/AIDS in 2014 in Uganda. Nakaseke district is bordered by Nakasongola district to the north ( Figure 1). The location of the district headquarters lies approximately 66 kilometers (41 mi) by road, north of Kampala, the capital of Uganda and the largest city in the country. It is estimated that 59.2% of the Nakaseke district community is literate, which is largely limited to the local Luganda language. This district has seven health units including a 100-bed public hospital, Nakaseke Hospital, administered by the Uganda Ministry of Health. Nakaseke Hospital is connected to other health units by a radio ^11^. One of the major health concerns is the high prevalence of HIV/AIDS. The prevalence rate of the disease in the district was estimated at about 8%, compared to the national average of 6.5%. Nakaseke district has the sixth-highest prevalence rate of HIV/AIDS in Uganda ^10^.

### Study population and sampling procedures

The sampling procedure was voluntary response participation. It was inexpensive since we needed to utilize students’ breakfast and lunch hours as requested by the school administration. Students from classes Senior 1, Senior 2, Senior 3, Senior 4, and Senior 6 were interviewed. Senior 5 students were excluded because they had not yet reported to school. The study was expected to interview a total of 200 students but only 163 of them turned up on the day of the study.

### Data collection

The students were interviewed using structured questionnaires ^12, 13^, with the help of trained research assistants in the students’ language of preference (English or Luganda). The questionnaire had closed-ended questions, presented in two formats: yes or no, and multiple choice or objective responses with an aim of accessing the knowledge of students about HIV/AIDS and their attitudes towards HIV transmission, prevention and stigma.

### Study variables

Treatment, transmission, prevention, cure, and diagnosis were the variables used to evaluate the degree of knowledge about HIV. This was done through computing percentages for every correct response and the KAP was categorised using Bloom's cut-off point where Bloom's cut-off of (≥80%) was used to determine sufficient knowledge. The demographic characteristics: age, gender and class were also considered.

### Data quality control

Trained research assistants interviewed the participants in their preferred language of communication (English or Luganda) to ensure comprehension and better expression while responding to the questions. Questions were simple and easy to understand. The data was entered using an excel spreadsheet, triple checked for errors, saved as a comma-separated values file (CSV) and later imported into R.

### Data analysis plan

All questions in the questionnaires were entered into the excel spreadsheet and the data was imported and analyzed using R version 3.6.2. All questionnaires with missing data were regarded as invalid and excluded from the analysis. The frequencies and percentage responses for each question was computed and the data was transformed into tables.

### Ethical considerations

Ethical clearance and approval for project was obtained from the School of Medicine Research and Ethics Committee (SOMREC) at Makerere University College of Health Sciences with approval number, #REC REF 2019–028. Administrative permission was obtained from both the Ugandan Ministry of Education and Sports (assigned reference number, ADM. 217/323/01) as well as the school. For participants in the age group 12 to 17 years, consent was sought from the school administrator and then assent was obtained from individual students. For mature and emancipated minors (between ages of 16 to 17), written informed consent was independently sought from them. For the age group of 18 years and above, consent was sought from the students themselves. Confidentiality of information was ensured by the principal investigator during and after the study by blinding the participants’ names and replacing them with arbitrarily chosen IDs.

## Results

163 participants volunteered for the study with more males (87, 53.37%) than females (76, 46.63%) ^14^. The participants were aged between 12 – 20 years, with a modal age group of 15 – 17 years and an average age of 15.45 ± 1.82.

In total, 143 (87.73%) participants were aware of HIV/AIDS while 20 (12.27%) were not. The mean age of those who were aware of the disease was 15.37 ± 1.77 years whereas those who were not had a mean age of 16.05 ± 2.06 years. The downstream analysis after the question regarding awareness of HIV/AIDS was exclusive of participants that were not aware of the disease. Most of the participants (109, 76.22%) were aware of HIV treatment (antiretroviral drugs; ARVs) while 34 (23.78%) were not aware. In total, 65 (60.75%) knew that HIV can be transmitted through sexual intercourse with an HIV infected person without using a condom, 5 (4.67%) mentioned sharing injecting equipment, and 68 had multiple responses (contaminated blood transfusion or organs/tissues, sexual intercourse with an HIV infected person without using a condom, sharing injecting equipment and mother to child transmission during childbirth and breastfeeding). Only 5 (4.67%) participants didn’t know of any mode of transmission ( Table 1). Blood (65, 53.72%) was the most known bodily fluid in which HIV is transmitted. Others mentioned breastmilk (1, 0.83%), vaginal fluids (4, 3.31%) and 33 (27.27%) participants did not know ( Table 2).

**Table 1.** Sources of information (multiple responses).

Response item	Response	Frequency	Percentage
**From which sources have you heard about HIV/AIDS**	Church or Mosque	2	1.92
	Family or Friends	20	19.23
	Health professionals	29	27.88
	TV or Radio	22	21.15
	Teachers or schools	31	29.81
**Total**		**104**	**100.00**
Response on modes of HIV/AIDS transmission			
**How is HIV transmitted**	Sexual intercourse without a condom	65	60.75
	Share injecting equipment	5	4.67
	Mother to Child transmission	27	25.23
	Contaminated blood transfusion	5	4.67
	Do not know	5	4.67
**Total**		**107**	**100.00**
Response to the cause of HIV/AIDS			
**What is the cause of HIV/AIDS**	Virus	88	61.54
	Bacteria	9	6.29
	Do not Know	46	32.17
**Total**		**143**	**100.0**

**Table 2.** Response on bodily fluids that contain HIV virus.

Response item	Response	Frequency	Percentage
**Which bodily fluids contain HIV**	Blood	65	53.72
	Breast milk	1	0.83
	Vaginal Fluids	4	3.31
	semen and rectal fluids or anal mucous	18	14.88
	Do not know	33	27.27
**Total**		**121**	**100.00**
Distribution of knowledge about HIV/AIDS patient			
**Signs and symptoms**	Frequent illness	105	73.43
	Do not Know	38	26.57
**Total**		**143**	**100.0**
**Have you ever seen HIV patient**	Yes	85	59.44
	No	58	40.56
**Total**		**143**	**100.0**
**If Yes, how did you Know**	Guessed	52	61.18
	Hospital	3	3.53
	Teachers/Schools	1	1.18
	Patient told you	26	30.59
	Family	3	3.53
**Total**		**85**	**100.01**

In regards to the cause of HIV/AIDS, most of the participants (88, 61.54%) knew that it was caused by a virus, 9 (6.29%) said that it was caused by bacteria and the rest 46 (32.17%) didn’t know the cause ( Table 1). For the signs and symptoms of HIV/AIDS, 105 (73.43%) individuals pointed out frequent illnesses and 38 (26.57%) did not know the signs and symptoms one could identify a person infected with HIV/AIDS. 85 (59.44%) participants had ever seen someone with HIV/AIDS whereas the rest 58 (40.56%) had never. Among those that had ever seen someone infected with the disease, 52 (61.18%) had guessed, 26 (30.59%) were told by the patient, 3 (3.53%) got to know through their families, 3 (3.53%) knew through the hospitals and 1 (1.18%) got to know through their teachers/schools ( Table 2).

When participants were asked whether there was a cure for HIV, 116 (81.12%) knew that there was no cure for HIV whereas 27 (18.88%) thought that there was a cure for HIV. Those that said there is a cure for HIV claimed to have heard from different sources of information. 5 (18.52%) from health professionals, 5 (18.52%) from friends/family, 8 (29.63%) from TV/Radio, 6 (22.22%) from Teachers/school, 2 (7.41%) from church/mosque and 1 (3.71%) from both health professionals and TV/ Radio. None got to know from the internet and newspapers ( Table 3). HIV prevention methods were generally known. Most responses (52, 46.02%) were for appropriate and consistent condom use, 48 (42.48%) were for abstinence, 2 (1.77%) for avoiding sharing sharp objects and the rest (1, 0.88%) were for taking prep consistently, checkups and avoiding blood contact with an infected person ( Table 3). Most of the participants (126, 88.11%) knew that HIV/AIDS was diagnosed using a blood test, 1 (0.70%) said that it was diagnosed through the urine test and 16 (11.19%) did not know how the disease is diagnosed ( Table 4).

**Table 3.** Response to the HIV cure presence.

Response item	Response	Frequency	Percentage
**Is there cure for HIV**	Yes	27	18.88
	No	116	81.12
**Total**		**143**	**100.00**
**If yes, how did you know about it**	Church or Mosque	2	7.41
	Family or Friends	5	18.52
	Health Professionals	5	18.52
	Radio or TV	8	29.63
	Teachers or schools	6	22.22
	Health professionals and TV	1	3.71
**Total**		**27**	**100.00**
Response to knowledge about HIV/AIDS prevention strategy			
**How do you prevent getting or** **transmitting HIV/AIDS**	Abstain	48	42.48
	Avoid sharing sharp objects	2	1.77
	Taking Prep or treatment consistently	1	0.88
	Checkups	1	0.88
	Do not know	7	6.19
	Appropriate condom use	52	46.02
	Avoid blood contact with infected person	1	0.88
	Testing for HIV/AIDS	1	0.88
**Total**		**113**	**100.00**

**Table 4.** Response on knowledge about HIV diagnosis.

Response item	Response	Frequency	Percentage
**How is HIV diagnosed**	Blood test	126	88.11
	Urine test	1	0.70
	Don’t know	16	11.19
**Total**		**143**	**100.00**
Response on knowledge of HIV infected parent to child transmission			
**One parent infected**	All	25	17.48
	Half	20	13.99
	Quarter	5	3.50
	None	79	55.24
	Do not know	14	9.79
**Total**		**143**	**100.00**
**All parents infected**	All	53	37.06
	Half	11	7.69
	None	60	41.96
	Do not know	19	13.29
**Total**		**143**	**100.00**

With regards to knowledge on HIV transmission from parent to child, most of the students were not sure whether the children would get infected in case at least one of the parents was HIV positive ( Table 4). Over two thirds (99, 69.23%) of students said they would not marry a person infected with HIV and 92 (69.70%) would discontinue the relationship if they discovered that the person they were dating was infected with HIV. Though 44 (30.77%) respondents were willing to marry a person infected with HIV, 9 (6.82%) would continue the relationship and 28 (21.21%) would seek counselling and treatment ( Table 5).

**Table 5.** Response on knowledge about dealing with HIV partners.

Response item	Response	Frequency	Percentage (%)
**Can you marry an HIV patient**	Yes	44	30.77
	No	99	69.23
**Total**		**143**	**100.00**
**What happens when you discover** **you are dating an HIV patient**	Continue with the relationship	9	6.82
	Discontinue the relationship	92	69.70
	Seek counselling and treatment	28	21.21
	Do not know	3	2.27
**Total**		**132**	**100.00**

Concerning knowledge on Host Genetics in HIV infection, 91 (63.64%) students had heard about the terms “DNA” or “Genes” and 52 (36.36%) hadn’t. For those who had heard about the terms, their major sources of information included; TV or radio (33, 38.37%), health professionals (29, 33.72%), family/friends (11, 12.79%), teachers/schools (11, 12.79%), church/mosque (1, 1.16%) and movies (1, 1.16%). Those who knew about DNA or genes were further asked whether their DNA or genes would determine if they would get infected with HIV or not; 56 (61.54%) said no, 31 (34.07%) said yes and 4 (4.40%) did not know. In addition, they were also asked whether their DNA or Genes would affect the outcome of their HIV treatment; 60 (65.93%) responded no to the question, 26 (28.57%) knew that DNA would affect HIV treatment whereas 5 (5.50%) were undecided ( Table 6).

**Table 6.** Response on knowledge on host genetics in HIV infection.

Response item	Response	Frequency	Percentage (%)
**Aware of the term DNA/genes**	Yes	91	63.64
	No	52	36.36
**Total**		**143**	**100.00**
**If yes, how did you hear them**	Church or Mosque	1	1.16
	Friends or Family	11	12.79
	Health professionals	29	33.72
	Teachers or schools	11	12.79
	TV or Radio	33	38.37
	Movies	1	1.16
**Total**		**86**	**100.00**
**Is HIV infection DNA determined**	Yes	31	34.07
	No	56	61.54
	Do not know	4	4.40
**Total**		**91**	**100.00**
**Does your DNA determine the** **outcome of your HIV treatment**	Yes	26	28.57
	No	60	65.93
	Do not know	5	5.50
**Total**		**91**	**100.00**

## Discussion

### Demographic characteristics

The study assessed the knowledge, attitudes and perception of secondary school students in Nakaseke, Uganda. Of the 163 participants, 143 (87.73%) were aware of HIV/AIDS and 20 (12.27%) were not. The mean age of those who were aware of the disease was 15.37 ± 1.77 years while those who were not aware of the disease had a mean age of 16.05 ± 2.06. Similar results were seen in other studies ^15, 16^

### Knowledge and attitude about HIV/AIDS treatment by participants in a rural school

Since awareness hinges on the knowledge about HIV/AIDS, what our participants knew about HIV was very key. The source of information about HIV was also important as it conveyed the basic information about HIV/AIDS and in this study. Teachers or schools (29.81%), and health professionals (27.88%) were the most used avenues to convey the message to the participants ^17^. The Presidential Initiative on AIDS Strategy to Youth (PIASCY) started in 2001, and was introduced in all primary and post primary schools’ education curriculum ^18^. This program was designed to prevent the spread of HIV/ AIDS and to mitigate its impact on primary and post-primary education institutions in Uganda. It is no surprise that the participants received most information about HIV/AIDS through their teachers ^18^. None of the participants got information through the internet and newspapers. This is typical of rural secondary schools as such services do not reach rural areas ^19^. A substantial number of students (87.73%) were aware of HIV/AIDS and its treatment (76.22%) except for a few. The bodily fluids through which HIV is spread were also known, with blood taking the biggest share (53.72%) hence being in concordance with other studies ^20, 21^. In spite of that, there were some misconceptions where some students thought HIV is spread through saliva, tears, vomitus and pus. These need to be addressed as they can cause stigma and discrimination towards patients with HIV.

### Knowledge and attitude about HIV/AIDS cure, symptoms and diagnosis

A small number of students (18.88%) thought HIV/AIDS is curable. They might have mistaken the available drugs for having a curative effect. To remedy this, more emphasis should be put on the microorganism that causes HIV because then, they would know the gravity of the disease and that it is incurable. Quite a number of students (32.17%) didn’t know what causes HIV/AIDS and others (6.29%) thought HIV/AIDS was caused by bacteria. Concerning the signs and symptoms of HIV/AIDS, several students knew them, showing that HIV/AIDS is not a rare disease. Most (61.18%) have known that someone has HIV/AIDS through guessing, 30.59% told by a patient, 3.53% told by family, and 3.53% have seen them in hospitals. Although there are several means through which students have known peoples’ HIV status, it is unethical for health practitioners to disclose a patient’s status as his/her confidentiality is violated ^22^. Therefore, people should be encouraged to take an HIV/AIDS test despite the way someone looks.

### Knowledge and attitude about HIV/AIDS prevention and transmission

Despite a vast knowledge on HIV/AIDS prevention like abstinence (42.48%), appropriate and consistent use of a condom (46.02%) to mention but a few, modern methods like taking prep (0.88%) in case of accidental infection or rape and mother to child transmission were less known to students. This was reflected by the few students that mentioned taking prep and their responses concerning a child’s chances of getting HIV/AIDS if either or both of the parents were infected. There was a difference in the responses to chances of a child getting HIV/AIDS if only one or both parents were infected showing that the students do not quite know that a child can be born without HIV/AIDS irrespective of the parents’ HIV status. It is important to sensitize the population on the recent advances in HIV/AIDS such as those mentioned above as they greatly help to control transmission of the disease and thus reduce the number of people infected with HIV/AIDS ^13^. Most participants were not willing to marry someone infected with HIV however there was a small percentage (4.90%) of people that were willing to continue the relationship and seek counseling and treatment. Nonetheless, even those (2.80%) that would discontinue the relationship were willing to encourage their partner to seek counseling and treatment. Despite that, there was still a huge number (69.70%) of people that didn’t want to have a relationship with someone with HIV. Therefore, there is still a lot of work needed to be done to change the attitude of these people towards HIV patients due to limited knowledge about HIV/AIDs in schools in remote areas.

### Knowledge and attitude about the role of an individual’s genetics on HIV/AIDS in acquisition and effect on treatment

Despite more than half (63.64%) of the total number of participants being familiar with the terms DNA and genes, 34.07% knew these terms in accordance with HIV transmission and 65.93% were informed about the terms in terms of HIV treatment

### Limitations of the study

Having carried out the study during the time senior five-students hadn’t resumed school, it reduced the classes interviewed thus affecting our study indirectly. Inaccurate responses from some participants could have limited the study as well. This would have been due to their fear to disclose what they knew

## Conclusion

This study provides preliminary data from a country and region where current information on the knowledge of young adults about HIV/AIDS and their attitude toward infected persons are sparse. This study highlights the basic knowledge of HIV/AIDS among young students, modes of transmission, treatment and management; it also indicates that stigma about the disease and discrimination of affected individuals in society is common among students. The basic approach for control and prevention of HIV/AIDS remains prevention through better knowledge and awareness since an effective cure or vaccine is not yet available.

Generally, the secondary school students had a high level of awareness of HIV/AIDS. However, with regards to aspects such as the cause and modern prevention methods like taking prep and prevention of mother to child transmission were less known to them. Efforts to find a cure for HIV/AIDS are still in vain. Therefore, strong emphasis on up to date control and prevention methods should be implemented to fight the HIV/AIDS scourge.

## Data availability

### Underlying data

Figshare: N_Raw_data.xlsx. https://doi.org/10.6084/m9.figshare.14399453 ^14^.

This project contains the following underlying data:

- N_Raw_data.xlsx (Raw survey data)

### Extended data

Figshare: Questionnaire.docx. https://doi.org/10.6084/m9.figshare.14399393 ^12^.

This project contains the following extended data:

- Questionnaire.docx

Data is available under terms of the Creative Commons Zero "No rights reserved" data waiver (CC0 1.0 Public domain dedication).
