# Supplementary material for: Knowledge and attitude of secondary school students in Nakaseke, Uganda towards HIV transmission and treatment
Source: AAS Open Res. 2021 Jul 12;4:23. Originally published 2021 May 5. [Version 2] doi: 10.12688/aasopenres.13210.2 (PMC8370127; doi:10.12688/aasopenres.13210.2)
Supplement: Peer review history

# Peer review: Reviewer response for version 2

**DOI:** 10.21956/aasopenres.14393.r28741

**Published:** 2021-08-16

**Authors:** Nchangwi Syntia Munung, Evelyn Ngwa Lumngwena

**Affiliations:** 1 Faculty of Health Sciences, University of Cape Town, Cape Town, South Africa

I have no further comments.

Is the work clearly and accurately presented and does it cite the current literature?

Partly

If applicable, is the statistical analysis and its interpretation appropriate?

Yes

Are all the source data underlying the results available to ensure full reproducibility?

Yes

Is the study design appropriate and is the work technically sound?

Partly

Are the conclusions drawn adequately supported by the results?

Yes

Are sufficient details of methods and analysis provided to allow replication by others?

Yes

Reviewer Expertise:

Two reviewers are involved and their areas of research include 1) Ethical legal and social Issues in Health research. 2) Infectious disease (HIV and RHD)-immunology and proteomics.

We confirm that we have read this submission and believe that we have an appropriate level of expertise to confirm that it is of an acceptable scientific standard.

# Peer review: Reviewer response for version 2

**DOI:** 10.21956/aasopenres.14393.r28742

**Published:** 2021-07-19

**Authors:** Vincent Mubangizi

**Affiliations:** 1 Mbarara University of Science and Technology, Mbarara, Uganda

Thank you for submitting the revised version.

Is the work clearly and accurately presented and does it cite the current literature?

Partly

If applicable, is the statistical analysis and its interpretation appropriate?

Not applicable

Are all the source data underlying the results available to ensure full reproducibility?

Yes

Is the study design appropriate and is the work technically sound?

Partly

Are the conclusions drawn adequately supported by the results?

Partly

Are sufficient details of methods and analysis provided to allow replication by others?

Yes

Reviewer Expertise:

Primary health care, Family planning, HIV/AIDS and dementia

I confirm that I have read this submission and believe that I have an appropriate level of expertise to confirm that it is of an acceptable scientific standard.

# Peer review: Reviewer response for version 1

**DOI:** 10.21956/aasopenres.14327.r28570

**Published:** 2021-05-27

**Authors:** Nchangwi Syntia Munung, Evelyn Ngwa Lumngwena

**Affiliations:** 1 Faculty of Health Sciences, University of Cape Town, Cape Town, South Africa

This manuscript describes the results of a survey on “Knowledge of HIV transmission and treatment amongst School Children in the Nakaseke district, Uganda”. As reported by the authors, there is a rise in new HIV cases in Uganda especially amongst young people, and it is therefore critical to gain an understanding of current knowledge on HIV transmission and care, in that population group. The results may inform HIV health promotion and education activities in the region. Below, for the authors’ consideration, are some suggestions for improving the manuscript.

**Major comments:**

1. The introduction needs more information to give context to readers on the current state of HIV awareness in Uganda, existing educational programs for youths and secondary school students in Uganda, generally and in schools in the Nakaseke district; and the potential of this study to inform HIV educational programs in Nakaseke and/or Uganda generally. Some of this information is in the discussion, but should have come earlier on in the introduction to provide the reader with some context on HIV awareness and education in Uganda and why it was important for the researchers to carry out this study.
2. The title of the questionnaire suggests that the study was on students’ awareness of host genomics and anti-retrovirals. This is an important and topical issue and one that has been less studied. However, in reporting the results of the study, less emphasis is given on this aspect of the study, including why it was important to survey students’ knowledge on host genomics and anti-retrovirals and if students had previously been exposed to such information, either as part of public health awareness programs, research, or educational programs in school. This should ideally be covered in the introduction and results section. Currently the main information is that the low awareness on HIV-host genomics and anti-retroviral therapy is not alarming as genomics is not thought in secondary schools. If that is the case, and that information was known, why did this section form part of the study?

**Methods:**

1. It is mentioned that the Nakaseke district was purposely selected because it has the sixth-highest prevalence rate of HIV/AIDS in Uganda. What is not clear is why the 1 ^st^ five districts with the highest prevalence were not selected or if similar studies have been carried out in this region. This information needs to come out clearly in the methods section, otherwise the justification for the study site is not convincing.
2. It is stated that sampling was voluntary. Kindly include a sentence, or two, and references, on what this sampling strategy involves and why it was used.
3. For readers who may not be familiar with the Ugandan school system, it is important to provide basic information on what S1 S2 is, in terms level of education (e.g. 5 years post primary, the age group etc) and possible HIV educational activities they may have been exposed to either in school, in their communities to suppose that this class is expected to have such knowledge etc.
4. The sentence “The prevalence rate of the disease in the district was estimated at about 8%, compared to the national aver-age of 6.5%”-needs a reference.
5. Kindly include more information on how the sample size of 200 students was reached, as well as what is meant by 163 students turn up for the study? Is this the number that consented, while the remaining 37 students did not? Or what were the reasons the other 37 did not complete the questionnaire? Information on how parental consent was obtained will be useful for including measures put in place to ensure that participation in the study was voluntary and that the students did not feel compelled to complete the study especially if it was administered in school.
6. Provide details on the administration of the questionnaire; was it done in school, or self-administered in some cases?
7. It is stated that the students were interviewed using structured questionnaires adopted from... (a reference is provided). For improved readability it is better to state what the questionnaire is and why that questionnaire was chosen over those that have been used in similar studies around the world. If any changes were made to the questionnaire, what were these changes and why was there a need to make these changes?

**Results:**

1. The results section has way too many tables (n=12) and this could distract from the main text. Some of the tables can be merged or deleted. For example, Table 1 and 2 can be deleted as that information has already been provided in the text in a clear fashion, therefore a table is not required. Also, some of the summary statistics in the tables could be deleted. Tables should have just key information for each response option rather than separately (option one and two). That is, it is better to provide percentages of people who selected abstinence, irrespective of whether that was selected together with another option, say appropriate condom use. The tables should give the reader an idea of how many persons ticked a particular response irrespective of whether the also selected another response. The methods already make it clear that in some cases participants selected more than one response. Table 3, 4 and 6 and 6 can be one table and should only contain information for each response not X and Y combined.
2. Table 7: The question “Have you ever seen an HIV patient” is ambiguous and could propagate stigma. The phrasing suggests that people with HIV look a specific way and can therefore be identified by merely looking at someone. That is disturbing for a study that looked at attitudes towards HIV. Was the question meant to be “Do you know of anyone living with HIV? If so, what was the purpose of the question - that information needs to be provided.
3. The referencing style and the way it is used in the article is very confusing, taking the reader away from the main text to the references. For example, page 8, (similar to 13) and page 10 (in concordance with 16 and 17). Our suggestion is that the authors should state the precise information they are referring to and then cite the text.

**Discussion and Conclusion:**

1. The discussion section could be shortened and streamlined to how the results obtained compare to similar studies in Uganda and globally and the implications that it may have for public health management of HIV/AIDS in Uganda. For example, one and two of the discussion section is a repetition of the results and that is the case for most of the paragraphs in that section. The authors repeat the results.
2. Given the low awareness of HIV/AIDS transmission, did the team introduce any educational intervention, pre or post survey? If so, what were these interventions and how was it received by the students? If not, are there plans to do awareness campaigns in the school - and what are the details particularly so on the role of host genomics in HIV transmission and therapy especially as this was the topic of the questionnaire? It will be a missed opportunity for a research program to take advantage of to raise awareness on HIV transmission and treatment especially as the authors note that there is rise in prevalence of HIV among young people in Uganda and that the district is one of those with the highest HIV prevalence in the country.

We thank the authors for sharing their results on this interesting study and look forward to reading their response to the comments that we have made.

Is the work clearly and accurately presented and does it cite the current literature?

Partly

If applicable, is the statistical analysis and its interpretation appropriate?

Yes

Are all the source data underlying the results available to ensure full reproducibility?

Yes

Is the study design appropriate and is the work technically sound?

Partly

Are the conclusions drawn adequately supported by the results?

Yes

Are sufficient details of methods and analysis provided to allow replication by others?

Yes

Reviewer Expertise:

Two reviewers are involved and their areas of research include 1) Ethical legal and social Issues in Health research .  2) Infectious disease ( HIV and RHD)-immunology and  proteomics

We confirm that we have read this submission and believe that we have an appropriate level of expertise to confirm that it is of an acceptable scientific standard, however we have significant reservations, as outlined above.

# Response

**Published:** 2021-07-03

**Authors:** Stephen Kanyerezi

**Affiliations:** Makerere University, Uganda

**Major comments:**

**The introduction needs more information to give context to readers on the current state of HIV awareness in Uganda, existing educational programs for youths and secondary school students in Uganda, generally and in schools in the Nakaseke district; and the potential of this study to inform HIV educational programs in Nakaseke and/or Uganda generally. Some of this information is in the discussion, but should have come earlier on in the introduction to provide the reader with some context on HIV awareness and education in Uganda and why it was important for the researchers to carry out this study.**

      

 We have enriched the introduction as suggested.

**The title of the questionnaire suggests that the study was on students’ awareness of host genomics and anti-retrovirals. This is an important and topical issue and one that has been less studied. However, in reporting the results of the study, less emphasis is given on this aspect of the study, including why it was important to survey students’ knowledge on host genomics and anti-retrovirals and if students had previously been exposed to such information, either as part of public health awareness programs, research, or educational programs in school. This should ideally be covered in the introduction and results section. Currently the main information is that the low awareness on HIV-host genomics and anti-retroviral therapy is not alarming as genomics is not thought in secondary schools. If that is the case, and that information was known, why did this section form part of the study?**

 The students did not have prior awareness of host genomics and antiretrovirals.

**Methods:**

**It is mentioned that the Nakaseke district was purposely selected because it has the sixth-highest prevalence rate of HIV/AIDS in Uganda. What is not clear is why the 1st five districts with the highest prevalence were not selected or if similar studies have been carried out in this region. This information needs to come out clearly in the methods section, otherwise the justification for the study site is not convincing.**

      

 We conveniently selected Nakaseke district because it was ranking poorly among in HIV/AIDS prevention

**It is stated that sampling was voluntary. Kindly include a sentence, or two, and references, on what this sampling strategy involves and why it was used.**

      

 We have included more details under Study population and sampling procedures.

**For readers who may not be familiar with the Ugandan school system, it is important to provide basic information on what S1 S2 is, in terms level of education (e.g. 5 years post primary, the age group etc) and possible HIV educational activities they may have been exposed to either in school, in their communities to suppose that this class is expected to have such knowledge etc.**

      

 These are classes or grades for Secondary level of education.

**The sentence “The prevalence rate of the disease in the district was estimated at about 8%, compared to the national aver-age of 6.5%”-needs a reference.**

      

 This was cited with citation 9.

**Kindly include more information on how the sample size of 200 students was reached, as well as what is meant by 163 students turning up for the study? Is this the number that consented, while the remaining 37 students did not? Or what were the reasons the other 37 did not complete the questionnaire? Information on how parental consent was obtained will be useful for including measures put in place to ensure that participation in the study was voluntary and that the students did not feel compelled to complete the study especially if it was administered in school.**

      

 The study school had a total of 200 students but 163 reported at school on the day of data collection.

**Provide details on the administration of the questionnaire; was it done in school, or self-administered in some cases?**

      

 It was administered at school within the school main hall, with help of research assistants to interpret to their local languages for those who could not comfortably read English and Luganda.

**It is stated that the students were interviewed using structured questionnaires adopted from... (a reference is provided). For improved readability it is better to state what the questionnaire is and why that questionnaire was chosen over those that have been used in similar studies around the world. If any changes were made to the questionnaire, what were these changes and why was there a need to make these changes?**

      

 Thank you for this comment, The questionnaire was purposely designed to probe aspects of knowledge and attitude of secondary school students in Nakaseke, Uganda towards HIV/AIDS transmission and treatment.

**Results:**

**The results section has way too many tables (n=12) and this could distract from the main text. Some of the tables can be merged or deleted. For example, Table 1 and 2 can be deleted as that information has already been provided in the text in a clear fashion, therefore a table is not required. Also, some of the summary statistics in the tables could be deleted. Tables should have just key information for each response option rather than separately (option one and two). That is, it is better to provide percentages of people who selected abstinence, irrespective of whether that was selected together with another option, say appropriate condom use. The tables should give the reader an idea of how many persons ticked a particular response irrespective of whether the also selected another response. The methods already make it clear that in some cases participants selected more than one response. Table 3, 4 and 6 and 6 can be one table and should only contain information for each response not X and Y combined.**

     We have addressed these accordingly as below; 

 Deleted table 1 and 2

 Merged tables 3, 4 and 6 into table 1

 Table 5, 7 merged into table 2

 Table 8, 9,  merged into table 3

 Table 10, 11 merged into table 4

 Table 12 to table 5

 Table 13 to table 6

**Table 7: The question “Have you ever seen an HIV patient” is ambiguous and could propagate stigma. The phrasing suggests that people with HIV look a specific way and can therefore be identified by merely looking at someone. That is disturbing for a study that looked at attitudes towards HIV. Was the question meant to be “Do you know of anyone living with HIV? If so, what was the purpose of the question - that information needs to be provided.**

    

 We agree. We meant to ask,  “Do you know of anyone living with HIV?

**The referencing style and the way it is used in the article is very confusing, taking the reader away from the main text to the references. For example, page 8, (similar to 13) and page 10 (in concordance with 16 and 17). Our suggestion is that the authors should state the precise information they are referring to and then cite the text.**

 This has been addressed accordingly.

**Discussion and Conclusion:**

**The discussion section could be shortened and streamlined to how the results obtained compare to similar studies in Uganda and globally and the implications that it may have for public health management of HIV/AIDS in Uganda. For example, one and two of the discussion section is a repetition of the results and that is the case for most of the paragraphs in that section. The authors repeat the results.**

      

 We have removed the repetition of the results.

**Given the low awareness of HIV/AIDS transmission, did the team introduce any educational intervention, pre or post survey? If so, what were these interventions and how was it received by the students? If not, are there plans to do awareness campaigns in the school - and what are the details particularly so on the role of host genomics in HIV transmission and therapy especially as this was the topic of the questionnaire? It will be a missed opportunity for a research program to take advantage of to raise awareness on HIV transmission and treatment especially as the authors note that there is rise in prevalence of HIV among young people in Uganda and that the district is one of those with the highest HIV prevalence in the country.**

 The post survey was not done due to school closure as a result of COVID-19 global Pandemic.

# Peer review: Reviewer response for version 1

**DOI:** 10.21956/aasopenres.14327.r28569

**Published:** 2021-05-24

**Authors:** Vincent Mubangizi

**Affiliations:** 1 Mbarara University of Science and Technology, Mbarara, Uganda

- **Is the work clearly and accurately presented and does it cite the current literature?**

**A.** The referencing style is confusing. For examples:

1. I could not trace the information attributed to citation 5.
2. Under the discussion section; similar results were seen in 11 and 12. …hence being in concordance with 16 and 17.  Similar to 13… etc Are these numbers referring to citations?
3. In the text, citation 9 is followed by citation 22. The same applies to citation 14 which is followed by citation 20.
4. I did not find citation 19 in the text.

**B.** Please provide current literature on HIV/AIDS prevalence in Uganda. The citation referring to the year 2014 is old. Citation 2 is wrongly cited to have provided information on the contribution of adolescents to HIV/AIDS prevalence globally yet that study was about knowledge, attitudes, and perceptions.

**C.** Under results section:

1. Many tables could be emerged to make it easy to read.
2. Since knowledge and attitudes are greatly influenced by the level of education, it would be better to indicate how the study participants were spread across the classes and if there was any difference in responses by the level of class attended.  
3. The statement “… *with regard to knowledge on HIV transmission from parent to child, the students’ response were generally not in line with the scientific facts*…” is not appropriate under this section.
4. Given that this was an interviewed administered tool and participants were in one place, where was there a high non-response rate? Could this affect the results?

**D. **Under the discussion section, the authors need to discuss how their results compare with the known literature.

1. Your finding of HIV/AIDS awareness is unique given the PIASCY program and that HIV/AIDS is a taught topic in the primary school curriculum. The government documents say that awareness is universal, Please discuss that key finding.
2. The second paragraph under Knowledge, attitudes about HIV/AIDS diagnosis … have many ideas. It would be better if they were separated. The assertion that “… *n this modern era, ARVs have made it increasingly difficult to identify someone living with HIV/AIDS “ *is misleading. When was it appropriate to make a diagnosis of HIV/AIDS by only physically looking at the person?
3. Under known and attitudes about the role of individual genetics, the assertion *“However, their knowledge of the terms was in respect to paternity tests rather than the role they play in acquisition and their effect on HIV/AIDS*” is not supported by data collected according to the data collection tool provided.

- **Is the study design appropriate and does the work have academic merit?**

The application of the results is affected by the lack of random sampling. The authors need to justify why they did not randomly sample the school and also why the “ *sampling procedure was voluntary*”. The authors need to show how they arrived at the number to be interviewed (sample size determination). These affect the generalizability of the study findings as well as introducing bias.

  

- **Are sufficient details of methods and analysis provided to allow replication by others?**

Under design and setting:

1. More information is required about the schools especially secondary schools in the study area; provide more information on the chosen school ( e.g ownership, day/boarding/ or both, school enrollment, etc).
2. For Figure 1; Use of Map tiler is protected under property intellectual rights, did the authors obtain permission to share the map in this publication?

Data collection:

1. It is mentioned that data was adopted from 10, provide a reference.
2. Also, specify if there was a translated version of the local language and how it was done to ensure consistency with the English version.
3. Was the questionnaire pre-tested?
4. Since according to the authors “ *questions were simple and easy to understand”,* why then did they use research assistants to interview participants.

Study variables:

1. It is not clear to me which study variable(s) was used to measure stigma.
2. Question 10 on the questionnaire seems to be hanging. It does not follow logically from questions 8 and 9.
3. The authors need to justify why they used a cut-off point of more than 50% to be knowledgeable about HIV/AIDS.

Data analysis:

1. Please indicate if any questionnaire (s) were excluded due to missing data.

Ethical considerations:

1. Please explain what you mean by “ *mature and emancipated minors*”.
2. Explain how reached the parents /guardians to sign consent forms for those aged 12-17 years.

- **If applicable, is the statistical analysis and its interpretation appropriate?**

Not applicable.

- **Are all the source data underlying the results available to ensure full reproducibility?**

Yes

- **Are the conclusions drawn adequately supported by the results?**

Partly. The conclusion about stigma about the disease and discrimination of affected individuals is not supported.

Is the work clearly and accurately presented and does it cite the current literature?

Partly

If applicable, is the statistical analysis and its interpretation appropriate?

Not applicable

Are all the source data underlying the results available to ensure full reproducibility?

Yes

Is the study design appropriate and is the work technically sound?

Partly

Are the conclusions drawn adequately supported by the results?

Partly

Are sufficient details of methods and analysis provided to allow replication by others?

Yes

Reviewer Expertise:

Primary health care, Family planning, HIV/AIDS and dementia

I confirm that I have read this submission and believe that I have an appropriate level of expertise to confirm that it is of an acceptable scientific standard, however I have significant reservations, as outlined above.

# Response

**Published:** 2021-07-03

**Authors:** Stephen Kanyerezi

**Affiliations:** Makerere University, Uganda

- **Is the work clearly and accurately presented and does it cite the current literature?**

**A. The referencing style is confusing. For examples:**

1. **I could not trace the information attributed to citation 5.**

Thank you. Citation 5 shows the current statistics of HIV under the tab “where we work”.

      

1. **Under the discussion section; similar results were seen in 11 and 12. …hence being in concordance with 16 and 17.  Similar to 13… etc Are these numbers referring to citations?**

Thank you. This has been corrected.

1. **In the text, citation 9 is followed by citation 22. The same applies to citation 14 which is followed by citation 20.**

Thank you. This has been corrected.

1. **I did not find citation 19 in the text.**

Thank you. This has been corrected.

**B. Please provide current literature on HIV/AIDS prevalence in Uganda. The citation referring to the year 2014 is old. Citation 2 is wrongly cited to have provided information on the contribution of adolescents to HIV/AIDS prevalence globally yet that study was about knowledge, attitudes, and perceptions.**

 Thank you. We have changed the citation to reflect the current HIV/AIDS prevalence in Uganda.

**C. Under results section:**

1. **Many tables could be merged to make it easy to read**.

           Thank you. We have merged the tables to make it easy to read

       

1. **Since knowledge and attitudes are greatly influenced by the level of education, it would be better to indicate how the study participants were spread across the classes and if there was any difference in responses by the level of class attended**. 

Thank you for this feedback however, we have excluded classes in the raw data as this would provide identifying information. This means we wouldn’t provide such information in the results/discussion.

1. **The statement “…***with regard to knowledge on HIV transmission from parent to child, the students’ responses were generally not in line with the scientific facts***…” is not appropriate under this section.**

We have replaced the above statement and reads, “most of the students were not sure whether the children would get infected in case at least one of the parents was HIV positive”

1. **Given that this was an interviewed administered tool and participants were in one place, where was there a high non-response rate? Could this affect the results?**

We agree that this tool was administered while participants were in one place but there was not a high non-response rate. We believe that this did not affect the results of this study.

**D. Under the discussion section, the authors need to discuss how their results compare with the known literature.**

1. **Your finding of HIV/AIDS awareness is unique given the PIASCY program and that HIV/AIDS is a taught topic in the primary school curriculum. The government documents say that awareness is universal, Please discuss that key finding.**

Our study in rural settings also confirmed the above fact as documented by the government of Uganda

1. **The second paragraph under Knowledge, attitudes about HIV/AIDS diagnosis … have many ideas. It would be better if they were separated. The assertion that “…***n this modern era, ARVs have made it increasingly difficult to identify someone living with HIV/AIDS “ ***is misleading. When was it appropriate to make a diagnosis of HIV/AIDS by only physically looking at the person?**
     
**“…** *n this modern era, ARVs have made it increasingly difficult to identify someone living with HIV/AIDS “ *Statement withdrawn 

We have separated the two ideas.

1. **Under known and attitudes about the role of individual genetics, the assertion ***“However, their knowledge of the terms was in respect to paternity tests rather than the role they play in acquisition and their effect on HIV/AIDS***” is not supported by data collected according to the data collection tool provided.**

We agree that the final tool administered did not have this section therefore the statement has been withdrawn.

- **Is the study design appropriate and does the work have academic merit?**

**The application of the results is affected by the lack of random sampling. The authors need to justify why they did not randomly sample the school and also why the “** *sampling procedure was voluntary* **”. The authors need to show how they arrived at the number to be interviewed (sample size determination). These affect the generalizability of the study findings as well as introducing bias.**

 We did not carry out random sampling but instead chose voluntary response participation because it was an inexpensive (no reimbursements) way to conduct this study in a school setting with minimum bias. However, we acknowledge that we could not have control over the makeup of the sample population that turned up to participate.

- **Are sufficient details of methods and analysis provided to allow replication by others?**

More information is required about the schools, especially secondary schools in the study area; provide more information on the chosen school ( e.g ownership, day/boarding/ or both, school enrollment, etc).

      

            We have included these details under design and setting:

1. **For Figure 1; Use of Map tiler is protected under property intellectual rights, did the authors obtain permission to share the map in this publication?**

Thank you, The source indicates that the citation is sufficient as long as it was not used for commercial purposes

**Data collection:**

1. **It is mentioned that data was adopted from 10, provide a reference.**

We have provided the reference.

1. **Also, specify if there was a translated version of the local language and how it was done to ensure consistency with the English version.** 

We did not translate the tool into the local language. The English version was piloted and deemed understandable to most secondary school level participants. 

1. **Was the questionnaire pre-tested?**

The questionnaire was pre-tested in the same school in 2019 prior to the execution of this current study, and included 25 students with a total of  5 students from each of the represented classes attending. During this study, students who had participated in the pretest were requested not to be part of the study. We have included these details in the revised manuscript under Study population and sampling procedures.

1. **Since according to the authors “***questions were simple and easy to understand”,*** why then did they use research assistants to interview participants.**

We used research assistants to interview participants to save on the students' school time. Since students were available during their breakfast and lunch time

**Study variables:**

1. **It is not clear to me which study variable(s) was used to measure stigma.**

This was a mistake on our part in the write-up. It has been replaced with cure and diagnosis

1. **Question 10 on the questionnaire seems to be hanging. It does not follow logically from questions 8 and 9.**

True, we agree that  the questionnaire on this section does not follow logically from questions 8 and 9.

      

1. **The authors need to justify why they used a cut-off point of more than 50% to be knowledgeable about HIV/AIDS.**

The level of KAP was categorized using Bloom's cut-off point where Bloom's cut-off of (≥80%) was used to determine sufficient knowledge. 

**Data analysis:**

1. **Please indicate if any questionnaire (s) were excluded due to missing data.**
       

           We did not exclude any questions due to missingness.

**Ethical considerations:**

1. **Please explain what you mean by “***mature and emancipated minors***”.**

Mature and emancipated minors referred to participants who are allowed to  some level of decision making such as adolescents who have the capacity to make decisions but who are not legally emancipated (between ages of 16 to 17). 

1. **Explain how reached the parents /guardians to sign consent forms for those aged 12-17 years.**

We have revised this section. The school administration signed on behalf of the parents /guardians under ethical considerations.
